# Bacterial Microcompartment-Mediated Ethanolamine Metabolism in Escherichia coli Urinary Tract Infection

**DOI:** 10.1128/IAI.00211-19

**Published:** 2019-07-23

**Authors:** Katherine Dadswell, Sinead Creagh, Edward McCullagh, Mingzhi Liang, Ian R. Brown, Martin J. Warren, Alan McNally, John MacSharry, Michael B. Prentice

**Affiliations:** a School of Microbiology, University College Cork, Cork, Ireland; b Department of Microbiology, Cork University Hospital, Cork, Ireland; c School of Biosciences, University of Kent, Canterbury, United Kingdom; d Institute of Microbiology and Infection, University of Birmingham, Birmingham, United Kingdom; e APC Microbiome Ireland, University College Cork, Cork, Ireland; f Department of Pathology, University College Cork, Cork, Ireland; University of California, Davis

**Keywords:** *Escherichia coli*, microcompartment, ethanolamine, metabolosome, urinary tract infection

## Abstract

Urinary tract infections (UTIs) are common and in general are caused by intestinal uropathogenic Escherichia coli (UPEC) ascending via the urethra. Microcompartment-mediated catabolism of ethanolamine, a host cell breakdown product, fuels the competitive overgrowth of intestinal E. coli, both pathogenic enterohemorrhagic E. coli and commensal strains. During a UTI, urease-negative E. coli bacteria thrive, despite the comparative nutrient limitation in urine. The role of ethanolamine as a potential nutrient source during UTIs is understudied. We evaluated the role of the metabolism of ethanolamine as a potential nitrogen and carbon source for UPEC in the urinary tract. We analyzed infected urine samples by culture, high-performance liquid chromatography, reverse transcription-quantitative PCR, and genomic sequencing. The ethanolamine concentration in urine was comparable to the concentration of the most abundant reported urinary amino acid, d-serine. Transcription of the *eut* operon was detected in the majority of urine samples containing E. coli screened. All sequenced UPEC strains had conserved *eut* operons, while metabolic genotypes previously associated with UTI (*dsdCXA*, *metE*) were mainly limited to phylogroup B2. *In vitro* ethanolamine was found to be utilized as a sole source of nitrogen by UPEC strains. The metabolism of ethanolamine in artificial urine medium (AUM) induced metabolosome formation and provided a growth advantage at the physiological levels found in urine. Interestingly, *eutE* (which encodes acetaldehyde dehydrogenase) was required for UPEC strains to utilize ethanolamine to gain a growth advantage in AUM, suggesting that ethanolamine is also utilized as a carbon source. These data suggest that urinary ethanolamine is a significant additional carbon and nitrogen source for infecting E. coli strains.

## Abstract (precis)

Urinary tract infections (UTIs) are common and in general are caused by intestinal uropathogenic Escherichia coli (UPEC) ascending via the urethra. Microcompartment-mediated catabolism of ethanolamine, a host cell breakdown product, fuels the competitive overgrowth of intestinal E. coli, both pathogenic enterohemorrhagic E. coli and commensal strains. During a UTI, urease-negative E. coli bacteria thrive, despite the comparative nutrient limitation in urine.

## INTRODUCTION

Urinary tract infection (UTI) is a common condition, with an estimated 150 million episodes occurring globally per annum (1). The most commonly identified cause is infection by uropathogenic Escherichia coli (UPEC) strains (2, 3). The currently accepted paradigm for uncomplicated urinary tract infection (UTI) is that these E. coli strains residing in the gut as commensals successively colonize the perineum (4), the urethra, and then the bladder, where the production of bacterial toxins and the host immune response lead to tissue damage and symptoms, such as frequency and dysuria (2). Further ascending infection to colonize the kidney with more local tissue damage, causing pyelonephritis and bacteremia, occurs in a small percentage of cases.

Common genetic features have been noted in a variety of E. coli strains causing infections outside the gastrointestinal tract, including UPEC strains, and these are collectively termed extraintestinal pathogenic E. coli (ExPEC) isolates (5, 6). Panels of genes whose presence is associated with any E. coli infection outside the gastrointestinal tract (7) or, specifically, urinary tract infection (8) have been assembled by genetic comparison of E. coli strains isolated from the gut with those isolated from urine and other extraintestinal sites and those known to be virulent in different animal models. However, the mechanism by which these factors are involved in pathogenicity is obscure.

In the pathogenesis of E. coli urinary tract infection, rapid invasion of bladder cells occurs with the formation of intracellular bacterial communities (IBCs) with biofilm-like properties, which initiate the infective process (9, 10). This bottleneck reduces diversity and has prevented global searches by signature-tagged mutagenesis for key genetic factors required for infection (11). Assessing genome-sequenced clinical E. coli urinary isolates in a mouse model of urinary tract infection showed that no set of genes was predictive of virulence in the model (12), including genes previously specifically associated with urovirulence.

Rapid growth has been shown to be characteristic of early-phase E. coli infection in the urinary tract (13), suggesting that securing nutrition in the urinary tract is a key part of E. coli pathogenesis. E. coli requirements for central carbon metabolism in the urinary tract have been explored by competition studies with selected mutants in murine models. Interruption of gluconeogenesis (*pckA*) or the tricarboxylic acid (TCA) cycle (*sdhB*) reduces the fitness of E. coli to infect (14). This is in contrast to the nutrient-rich intestine, where glycolysis (*pgi*) or the Entner-Doudoroff (*edd*) pathway is required for colonization fitness (15).

Some metabolic loci have been linked to UPEC pathogenesis. d-Serine is an abundant amino acid in human urine and is present at a mean concentration of 0.12 mM (16) and up to 1 mM in some cases (17), much higher than intestinal content levels. Some E. coli strains can metabolize d-serine to pyruvate and ammonia (18), allowing it to be a sole carbon and nitrogen source *in vitro* (19). This is conferred by possession of a complete d-serine tolerance locus (*dsdCXA*) (20), where *dsdC* encodes a LysR-type transcriptional regulator (LTTR), *dsdX* encodes a d-serine transporter (21), and *dsdA* encodes a d-serine dehydratase. ExPEC strains usually encode a full *dsdCXA* locus, while enteric pathogenic E. coli strains frequently have a truncation after *dsdC* (22). In the absence or truncation of this locus, d-serine shows reversible toxicity for E. coli, causing growth arrest at concentrations of 0.1 mM and above *in vitro* (23).

A metabolic regulatory polymorphism has been associated with cobalamin-independent methionine synthase (MetE) in UPEC. A promoter polymorphism (*sra* [short regulatory allele]) upstream of the *metE* gene in these strains is associated with increased *metE* induction and an enhanced ability to grow in urine *in vitro* (24).

Mutational analysis of a subset of E. coli genes showing a marked (greater than 4-fold) increase in transcription in infected patient urine compared to that with growth in urine or Luria broth (LB) (25) showed that that their knockout caused a fitness defect in the urinary bladder in a mouse model of ascending urinary tract infection. The most marked defects were with knockout of the *cus* (encoding copper resistance) and *eut* (encoding ethanolamine uptake and metabolism) operons.

**FIG 1:**
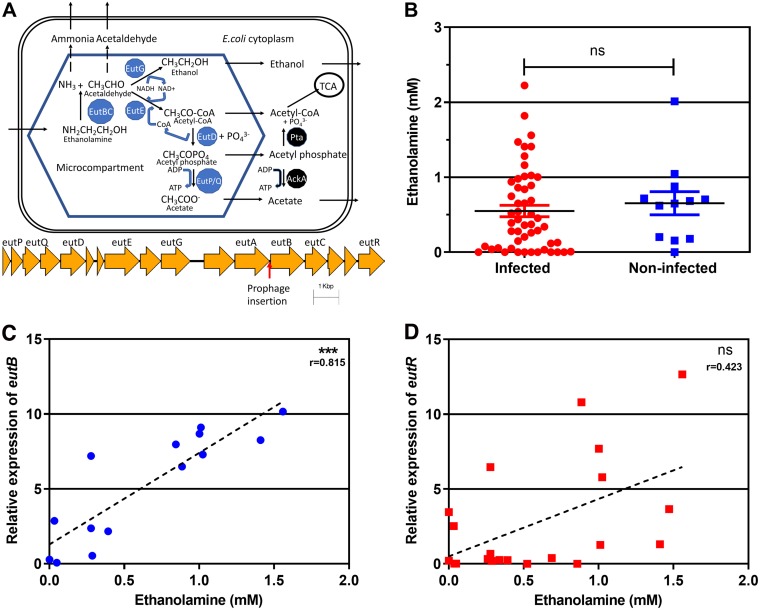
Ethanolamine is present in urine, and the urinary ethanolamine concentration correlates with the expression of *eut* operon genes in E. coli-infected urine. (A) Microcompartment-mediated ethanolamine metabolic pathway and *eut* operon. Black arrows, metabolite translocation or reaction; dotted arrows, metabolite translocation impeded by the microcompartment; blue hexagon, the microcompartment; blue, microcompartment-associated enzymes; black, cytoplasmic enzymes; yellow arrows, the *eut* operon; red arrow, prophage insertion hot spot. (B) Ethanolamine concentration in urine. There was no significant difference in the ethanolamine concentration between clinically infected urine samples and control noninfected samples (Mann-Whitney U test). (C) Correlation between the ethanolamine concentration in infected urine and expression of *eutB* (relative to that of *gyrA*) (***, *P* < 0.001). (D) Correlation between the ethanolamine concentration in infected urine and expression of *eutR* (relative to that of *gyrA*). *r*, Spearman's rank correlation coefficient; ns, not significant.

The *eut* operon is part of the conserved E. coli core genome (26), having arrived in *Enterobacterales* by horizontal transfer (27). It contains 17 genes, including the positive transcriptional regulator *eutR*. The operon encodes enzymes required for ethanolamine metabolism and includes structural shell protein genes for the synthesis of thin porous protein shells enclosing the enzymes as bacterial microcompartments (metabolosomes) in the cytoplasm (28–30) (Fig. 1A). Experiments largely conducted with Salmonella enterica (which contains the same operon) suggest that the enzymatic breakdown of ethanolamine to ammonia (a nitrogen source) and acetaldehyde occurs within the metabolosome, with the toxic effects and evaporative loss of acetaldehyde being minimized by microcompartment enclosure and onward metabolism to ethanol and acetyl coenzyme A (acetyl-CoA) (a carbon source) (30, 31). Some acetyl-CoA is further metabolized to acetyl phosphate and acetate within the metabolosome, and some is available to enter central metabolism (32). The ethanolamine in the gastrointestinal tract utilized by this pathway gives a competitive advantage to enterohemorrhagic E. coli (EHEC) (33) and Salmonella enterica serovar Enteritidis (34). Recently, it has been shown that E. coli ethanolamine metabolism is essential for bladder colonization in a murine model of ascending UTI (35). The mechanism was suggested to involve resistance to innate immunity because the colonization advantage of wild-type UPEC over a Δ*eutR* mutant was abolished in neutrophil-depleted mice. Clearance of an isogenic Δ*eutR* mutant E. coli strain from the bladder coincided with peaking myeloperoxidase levels. However, resistance to hydrogen peroxide was unchanged in the Δ*eutR* mutant.

In this study, we evaluated the role of microcompartment-mediated ethanolamine metabolism in clinically infected urine samples and in laboratory cultures of E. coli strains isolated from infected urine. The *eut* operon was induced in infected urine, and ethanolamine was present in urine at a level that enhanced E. coli growth *in vitro*. Metabolosomes were visible on transmission electron microscopy (TEM) in a UPEC strain grown with ethanolamine. Inactivation of the *eut* operon reduced the growth of a UPEC strain in ethanolamine-containing nitrogen-limited minimal medium and growth and competitiveness in ethanolamine-containing artificial urine medium (AUM). The selective mutation of individual *eut* genes suggested that ethanolamine provides a carbon source in this artificial urine medium. In summary, we have identified that microcompartment-mediated metabolism of the ethanolamine present in urine can give E. coli a growth advantage by providing an additional carbon and nitrogen source.

## RESULTS

### Ethanolamine is present in urine, and infecting E. coli strains show *eut* operon induction.

One hundred three clinically infected urine samples were selected. Sixty-one E. coli strains were isolated from these samples, and 47 of these were sequenced and used for *in vitro* metabolic analysis. The mean ± standard error of the mean (SEM) concentration of ethanolamine was 0.55 ± 0.076 mM in 54 clinically infected urine samples and 0.66 ± 0.155 mM in 12 control urine samples which were not clinically infected (they contained no white cells or bacteria on microscopy) (Fig. 1B). The difference between infected and control urine samples was not significant. In 24 E. coli-infected urine samples from which RNA was extracted, transcription of *eut* operon genes was detected in the majority of cases for *eutB* (88%), *eutS* (68%), and *eutR* (63%). Expression of *eutB* significantly correlated with the ethanolamine concentration in urine (Fig. 1C), while expression of *eutR* did not (Fig. 1D). Because of anonymization, individual patient details were not available. An audit of all diagnostic urine specimens in the Cork University Hospital microbiology laboratory in 2018 shows that 75% come from general practice, 25% come from hospital sources, and 75% overall come from women.

### Clinically infected urine samples show stimulation of the host innate immune response.

The cytokines interleukin-8 (IL-8) and IL-1β were detected in 81% of the clinically infected urine samples, and their levels were significantly increased in infected urine samples compared to noninfected urine samples (for IL-1β, *P* = 0.0048; for IL-8, *P* < 0.001; see Fig. S1 in the supplemental material). Mean IL-6 levels were higher in infected urine than in noninfected urine, but the difference was not significant (Fig. S1).

### Uropathogenic E. coli strains utilize ethanolamine *in vitro*, resulting in enhanced growth, formation of bacterial microcompartments, and production of acetate and ethanol.

**FIG 2:**
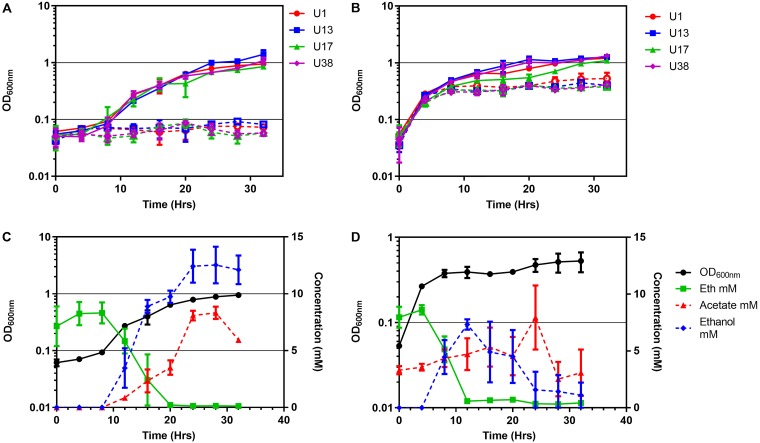
Ethanolamine metabolism promotes UPEC growth in nitrogen-limited minimal medium and artificial urine medium (AUM). (A and B) Aerobic growth of selected UPECs at 37°C in ammonia-free modified M9 medium with glycerol (20 mM) (A) or in AUM (B). Hollow data points are without ethanolamine; solid data points are with an additional 10 mM ethanolamine. (C and D) Concentrations of ethanolamine (Eth), acetate, and ethanol over time during U1 growth in ammonia-free M9 medium with glycerol (20 mM) (C) and AUM (D), both of which were supplemented with an initial 10 mM ethanolamine. Values are the mean ± SEM (*n* ≥ 3).

**FIG 3:**
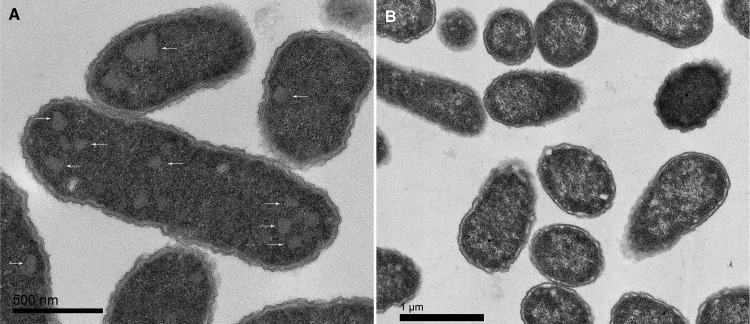
Growth of UPEC strain U1 in artificial urine medium with ethanolamine promotes the formation of bacterial microcompartments. Transmission electron microscopy was performed following culture for 8 h in AUM with 10 mM ethanolamine (A) or in AUM alone (B). White arrows indicate microcompartments.

Forty-five out of 47 (96%) E. coli strains isolated from urine showed increased overnight growth with 10 mM ethanolamine as the sole nitrogen source in M9 minimal medium (Fig. S2). No increased growth was detected with 10 mM ethanolamine as a sole carbon source in M9 medium for four strains shown to actively metabolize ethanolamine as a nitrogen source (Fig. S2 and S3). For these selected strains (strains U1, U13, U17, U38), growth in M9 medium with ethanolamine containing glycerol as a carbon source commenced after 10 h (Fig. 2A), with ethanolamine consumption occurring from about 8 h (Fig. 2C). Addition of 10 mM ethanolamine to artificial urine medium (AUM) also increased the growth of these strains (Fig. 2B), with the consumption of ethanolamine occurring from about 4 h of incubation onwards (Fig. 2D). Acetate and ethanol were produced by E. coli U1 grown in both M9 medium and AUM when ethanolamine was added (Fig. 2C and D) and corresponded to induction of the *eut* operon at 4 and 8 h of incubation, respectively, with ethanolamine in AUM (Fig. S4). TEM of E. coli U1 grown in AUM with added ethanolamine showed 100- to 130-nm cytoplasmic inclusions with straight edges (Fig. 3A) in the majority of cells visualized (43/69 [62%]). These structures are typical of bacterial microcompartments. They were not observed in cells grown in the absence of ethanolamine (Fig. 3B) and were seen in a minority of cells grown in minimal medium with ethanolamine (Fig. S5). The difference in TEM appearances between M9 medium and AUM may be growth phase related. Cells were collected for TEM at 8 h of incubation, which is approximately the starting time for ethanolamine consumption in M9 minimal medium but the time of the most rapid consumption in AUM (Fig. 2). Acetate was detected in nearly all infected urine samples tested (Fig. S8).

### The effect of inactivation of individual enzyme-encoding genes in the *eut* operon suggests that ethanolamine growth stimulation in artificial urine medium is due to provision of an additional carbon source.

Mutation of the *eut* operon genes *eutB* and *eutE* was achieved in strain U1 (Table 1). *eutB* encodes the heavy-chain component of ethanolamine ammonia lyase, required to liberate ammonia from ethanolamine, and *eutE* encodes a reversible acetaldehyde dehydrogenase, acting after *eutBC* in the ethanolamine catabolism pathway (see the schematic in Fig. 1A). EutE is required to generate acetyl-CoA, which is the route for carbon assimilation from ethanolamine (Fig. 1A).

**TABLE 1:** Plasmids and strains in this study

Plasmid or strain	Genotype/designation	Source (reference)
Plasmids		
pCA24N	High-copy-number expression vector, *cat*	NBRP E. coli, Japan (68)
pCA24N::*eutB*	ASKA clone JW2434	NBRP E. coli, Japan (68)
pCA24N::*eutE*	ASKA clone JW2439	NBRP E. coli, Japan (68)
Strains		
E. coli U1	E. coli phylogroup A urine isolate	This study
E. coli JW2434-1	BW25113 *ΔeutB*	Keio Collection, Japan (66)
E. coli JW2439-1	BW25113 *ΔeutE*	Keio Collection, Japan (66)
E. coli U1 *ΔeutB*	*ΔeutB*::*kan*	This study
E. coli U1 *ΔeutE*	*ΔeutE*::*kan*	This study
U2 to U79 (46 strains)	E. coli urine isolates	This study

**FIG 4:**
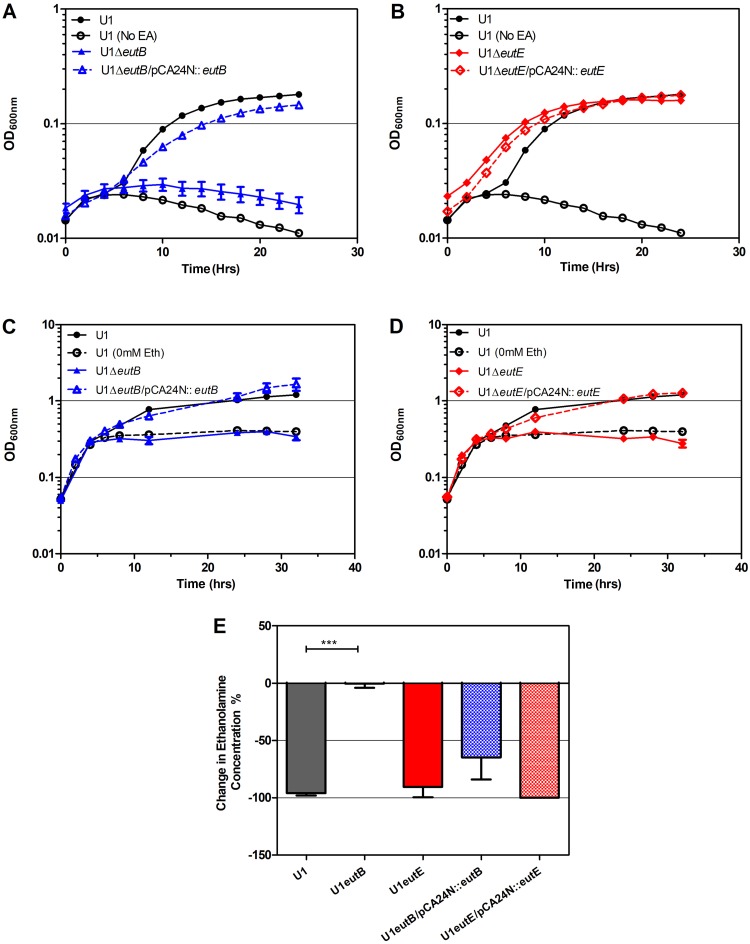
*eutE* inactivation in UPEC strain U1 abolishes ethanolamine growth stimulation in AUM, despite preserved ethanolamine catabolism. (A) Growth of U1, the U1 Δ*eutB* mutant, and the complemented strain in modified M9 plus 10 mM ethanolamine. (B) Growth of U1, the U1 Δ*eutE* mutant, and the complemented strain in modified M9 with 10 mM ethanolamine. (C) Growth of U1, the U1 Δ*eutB* mutant, and the complemented strain in AUM plus 10 mM ethanolamine. (D) Growth of U1, the U1 Δ*eutE* mutant, and the complemented strain in AUM with 10 mM ethanolamine. In panels A to D, the growth of U1 in control medium without ethanolamine (No EA or 0 mM Eth) is shown as open circles. (E) Percent change in ethanolamine concentration measured by HPLC over 24 h of U1, U1 Δ*eutB*, U1 Δ*eutE*, and their complemented strains in AUM with an initial 10 mM ethanolamine. ***, significant difference from the wild type (*P* < 0.001, 1-way analysis of variance). All values are the mean ± SEM (*n* = 3).

Growth stimulation in nitrogen-limited minimal (M9) medium by addition of ethanolamine (0.5 mM or 10 mM) was abolished by deletion of *eutB* in U1 and retained after deletion of *eutE* (Fig. 4A and B; Fig. S6; Table 2). Reverse transcription-PCR (RT-PCR) showed that ethanolamine induced *eutE* transcription in the *eutB* mutant and vice versa, demonstrating that these were not polar mutations (Fig. S4). Ammonia generation from ethanolamine alone is therefore sufficient to stimulate E. coli U1 growth in nitrogen-limited minimal (M9) medium. Complementation of the *eutB* mutant restored the wild-type phenotype in ethanolamine-containing minimal medium (Fig. 4A).

**TABLE 2:** *In vitro* growth phenotype of wild-type strain U1 and *eut* operon mutants with additional ethanolamine

Genotype	Growth phenotype after growth in^*a*^:			
	M9 with 10 mM Eth	M9 with 0.5 mM Eth	AUM with 10 mM Eth	AUM with 0.5 mM Eth
U1 wild type	+	+	+	+
U1 Δ*eutB*	−	−	−	−
U1 Δ*eutE*	+	+	−	−
U1 Δ*eutB/*pCA24N::*eutB*	+	+	+	ND
U1 Δ*eutE/*pCA24N::*eutE*	+	+	+	ND

a +, growth enhancement compared to growth without ethanolamine; −, no growth enhancement compared to growth without ethanolamine; ND, no data; Eth, ethanolamine; M9, minimal medium; AUM, artificial urine medium.

In contrast to this phenotype in nitrogen-limited minimal (M9) medium, in AUM, which contains 25 mM ammonium chloride and no glycerol as a carbon source, growth stimulation by ethanolamine was absent in U1 Δ*eutE*, although ethanolamine was still metabolized by this strain (Fig. 4D and E; Table 2). Growth enhancement by ethanolamine in AUM was restored by *eutE* complementation. In AUM, U1 Δ*eutB* showed no growth stimulation by ethanolamine and no ethanolamine metabolism, both properties being restored by *eutB* complementation (Fig. 4C and E; Table 2). Therefore, in AUM, unlike nitrogen-limited M9 medium, the growth stimulation conferred by ethanolamine metabolism is not due to ammonia generation but appears to be caused by the provision of an additional carbon source from acetyl-CoA.

### A functional *eut* operon is essential for competitive growth of a UPEC strain in the presence of ethanolamine *in vitro*.

**FIG 5:**
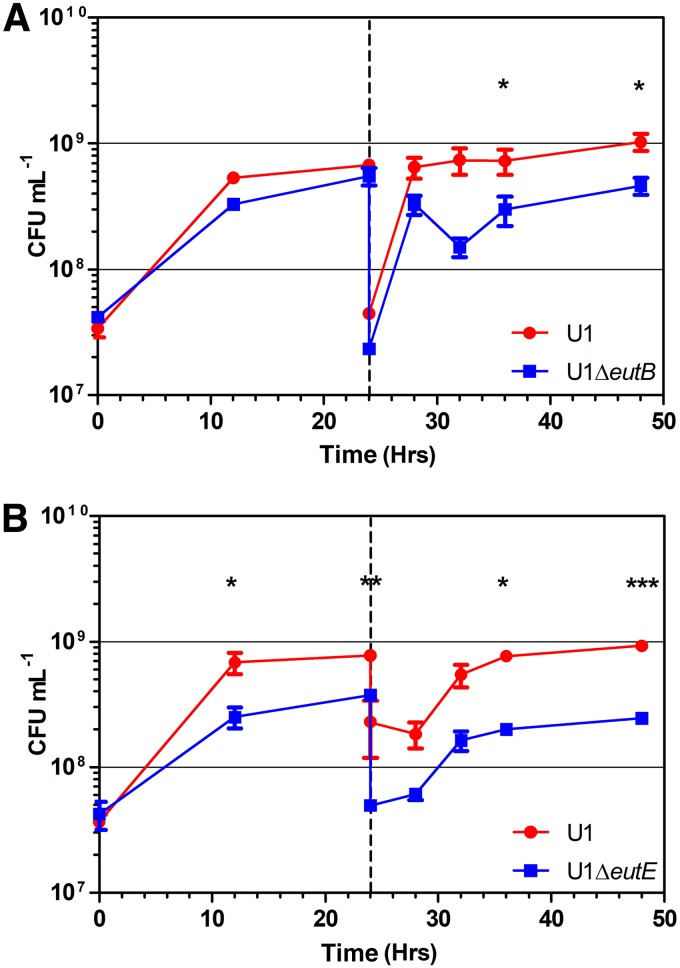
Inactivation of the *eut* operon genes reduces the competitiveness of uropathogenic E. coli strain U1 in artificial urine medium containing 10 mM ethanolamine. The competition of U1 versus U1 Δ*eutB* with 10 mM ethanolamine (A) or U1 Δ*eutE* with 10 mM ethanolamine (B) is shown. Values are the mean ± SEM (*n* = 3). *P* values were determined by the Mann-Whitney U test. *, *P* < 0.05; **, *P* < 0.01; ***, *P* < 0.001.

Competitive growth assays in AUM containing 10 mM ethanolamine between wild-type E. coli strain U1 and the Δ*eutB* and Δ*eutE* mutants showed a significant advantage for the wild type after 32 h (incorporating a 24-h subculture) for both mutants (Fig. 5). The Δ*eutE* mutant showed a significant disadvantage from 12 h onwards. The competitive index (CI) of both mutants at all time intervals from 12 h onwards was less than 0.8 (Table S2). No significant difference in competitive growth between the wild type and the mutants was found in AUM with 0.5 mM ethanolamine (Fig. S7) or in the absence of ethanolamine (data not shown).

### The *eut* operon is conserved in all UPEC strains sequenced, while putative urovirulence factors and metabolic polymorphisms previously associated with UPEC are phylogroup related.

**FIG 6:**
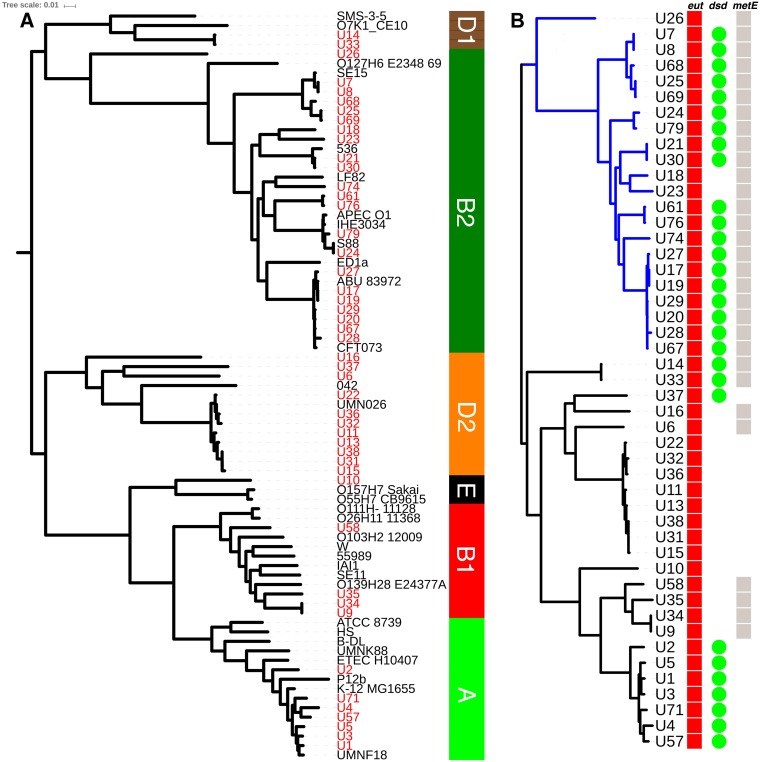
Phylogenetic distribution of E. coli urine isolates from this study and the conservation of metabolic operons. (A) The phylogeny of 47 strains (taxon labels in red) isolated from infected urine analyzed by core genome alignment, using Parsnp, with 32 reference strains representative of six E. coli phylogroups (taxon labels in black). Bootstrap values for all internal nodes were 1.0 apart from the node (0.25) between the reference strains APECO1 and IHE3034, which constitute the least-diverged core genome pair in the reference set. Clade assignments are shown in the vertical bar on the right. (B) Parsnp alignment of the 47 strains alone. The B2 phylogroup is colored blue. Vertical bars/circles indicate the presence of a complete *eut* operon (red), a complete *dsdCXA* locus (green), and a short regulatory *metE* allele (gray) in each strain.

A single nucleotide polymorphism (SNP)-based tree from a core genome alignment of the 47 urine E. coli isolates and 32 representative reference strains by Parsnp (36) assigned all urine strains to phylogroups (Fig. 6). The largest single grouping of urine E. coli isolates was formed by 22 phylogroup B2 strains (46%) (Fig. 6), followed by 11 phylogroup D2 (23%), 7 phylogroup A (15%), 4 phylogroup B1 (9%), and 2 phylogroup D1 (4%) strains and 1 phylogroup E (2%) strain. The tree shown used U7 from this study as the reference strain for SNPs, and the core 79-genome alignment (47 from this study plus 32 phylogroup representatives) included 53% of the U7 genome. The same phylogroup assignments were found in trees generated with finished closed GenBank genome sequence strains from each phylogroup as the SNP reference strain, as expected (36).

**FIG 7:**
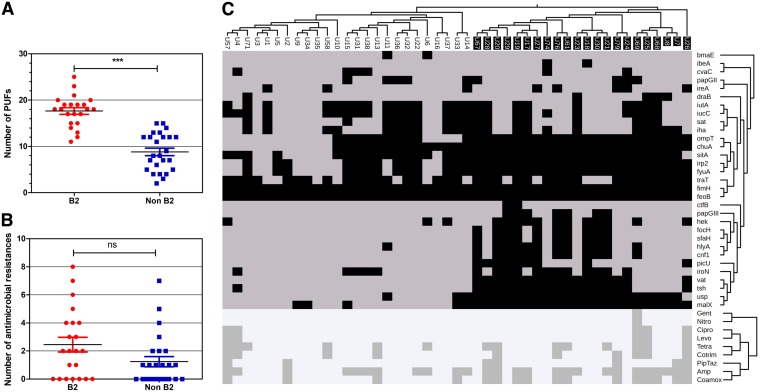
Carriage of putative virulence factors (PUFs) but not antimicrobial resistance is associated with clade B2 E. coli urine isolates. (A) PUF scores differ between the B2 and non-B2 groups. ***, *P* < 0.0001, Mann-Whitney U test. (B) The antimicrobial resistance scores (number of different antimicrobials to which the strain is resistant) do not differ between the B2 and non-B2 groups. (C) The genome sequences of clinical urine E. coli isolates were screened for the presence of 31 previously described PUFs (*y* axis labels) using BLASTN analysis. The presence (black squares) or absence (gray squares) is shown for each PUF in relation to each isolate. Two-dimensional hierarchical clustering shows the PUF cooccurrence by strain (upper *y* axis dendrogram) and the PUF association with the phylogeny (*x* axis dendrogram). Clade B2 strains are indicated by white names on a black background (*x* axis labels). The lower diagram shows the hierarchical clustering of resistance (dark gray squares) and sensitivity (pale gray squares) to nine different antimicrobials (lower *y* axis dendrogram) by strain phylogeny. Abbreviations: Gent, gentamicin; Nitro, nitrofurantoin; Cipro, ciprofloxacin; Levo, levofloxacin; Tetra, tetracycline; Cotrim, co-trimoxazole; PipTaz, piperacillin-tazobactam; Amp, ampicillin; Coamox, amoxicillin-clavulanic acid.

The presence of a set of 31 previously described (12) putative virulence factors (PUFs) determined by a BLASTN search was used to score each of the 47 E. coli genomes. These represented a compilation of genes previously found to be enriched in UTI E. coli strains compared to other E. coli strains (37–40). All 31 PUFs were found in the set of genomes, and the median PUF count was 13 (range, 2 to 25). Phylogroup B2 E. coli urine isolates had higher PUF counts than non-B2 strains (*P* < 0.001, Mann-Whitney U test) (Fig. 7A). Hierarchical clustering of the PUF carriage profiles showed PUF profile patterns related to B2 clade membership (Fig. 7C), while clustering of antimicrobial resistance phenotypic profiles showed no obvious phylogenetic relationship (Fig. 7B).

Regarding metabolic features, the *eut* operon was conserved in all 47 strains (Fig. 6B). However, strain U71 contained a novel prophage in the same site as the CPZ-55 prophage insertion between *eutA* and *eutB* characteristic of E. coli MG1655 (41) and other K-12 lineage strains (Fig. 1A). Genome sequencing of the knockout strains U1 *ΔeutB* and U1 Δ*eutE* (see above) revealed the expected single-gene deletions (marked by a kanamycin resistance cassette).

A short *metE* regulatory allele was present in 30 strains, and a complete d-serine tolerance locus (*dsdCXA*) was present in 29 strains (Fig. 6B). All strains contained a complete *yhaOMKJ* d-serine sensory locus. B2 strains were more likely to possess a short *metE* regulatory allele and a complete *dsdCXA* locus than non-B2 strains (2-sided *P* < 0.0001 and *P* < 0.0022, respectively, Fisher's exact test).

## DISCUSSION

The E. coli strains isolated from urine in this study were phylogenetically similar to strains of previously published urinary tract infection series, in that B2 and D2 were the two most common phylogroups (42). We report a lower proportion of B2 strains (46%) (Fig. 6) than that reported by urosepsis and urinary tract infection studies from the United States and Spain (67% to 69%) (12, 40, 43, 44), a proportion similar to that reported in Slovenia (50%) (45), and a proportion greater than that reported in Denmark (34%) (46) and China (19%) (42). The PUF profile association demonstrated with phylogroup B2 (Fig. 7) is consistent with previous findings from a set of urinary tract infection isolates from the United States (12). This study found that B2 strains not associated with urinary tract infection are also enriched for these genes and that the PUF profile does not correlate with virulence in animal models of UTI (12). Phylogroup B2 strains are more likely than strains of other phylogroups to colonize the gut (47, 48), and these putative urovirulence factors may in fact be more important in the gut. Similarly, we found that the metabolic loci proposed to be helpful for growth in urine, such as d-serine tolerance and a short *metE* allele, were also associated with phylogroup B2 (Fig. 6).

In contrast, the *eut* operon was conserved in all isolates (Fig. 6), and the ability to utilize ethanolamine *in vitro* was observed in 96% of strains (see Fig. S2 in the supplemental material). This is not surprising, because the E. coli core genome includes the *eut* operon (26). Therefore, the presence of ethanolamine accessible in urine is potentially a significant nutritional resource for all phylogroups of UPEC.

We found similar concentrations of ethanolamine in infected urine from patients (mean ± SEM, 0.55 ± 0.076 mM) and noninfected urine controls (mean ± SEM, 0.66 ± 0.155 mM) (Fig. 1B). The levels are consistent with those from previous reports on smaller numbers of samples from healthy controls determined using a different methodology, such as nuclear magnetic resonance (NMR) (0.38 mM) (49) and liquid chromatography/mass spectrometry (0.47 mM) (50). The NMR study found ethanolamine in all 22 urine specimens processed (49). The lack of ethanolamine in a minority of our infected specimens (9/54; Fig. 1B) may reflect the limitations of the high-performance liquid chromatography (HPLC) assay. The maximal ethanolamine concentration in bovine intestinal content (BIC; the filtered contents of the jejunum and ileum), where enterohemorrhagic E. coli has been shown to gain an *in vitro* competitive advantage by ethanolamine utilization, is 2.2 mM (33). For comparison, d-serine is regarded as an abundant substrate for E. coli metabolism in human urine (51), where it has been reported at a mean concentration of 0.12 mM out of a total mean urine serine concentration of 0.33 mM (16).

We found evidence that ethanolamine in infected urine was sensed by E. coli with induction of the *eut* operon regulator *eutR* and was being metabolized, with induction of the ethanolamine deaminase component *eutB* correlating with the ethanolamine levels measured in urine (Fig. 1C and D). *In vitro*, UPEC strains produced acetate and ethanol when metabolizing ethanolamine in both minimal medium and artificial urine medium (Fig. 2), as expected (Fig. 1A) (52). Acetate was also detected in infected urine (Fig. S8), as previously reported for infected urine samples with a variety of different bacterial causes of infection (53). Acetogenic growth of E. coli *in vivo* is hypothesized to be an essential property in urinary tract infection (54, 55) and has been ascribed to the metabolism of d-serine via pyruvate to acetyl-CoA and acetyl phosphate (54, 56). We propose that the consistent presence of host-derived ethanolamine in urine at higher concentrations than d-serine also contributes to this phenotype. Acetate is an important regulator of E. coli gene expression (56) and the host immune response (57) and may contribute to the previously reported (35) phenotype linking the *eut* operon to resistance to innate immunity.

TEM revealed that cells metabolizing E. coli *in vitro* in AUM produced numerous plane-edged cytoplasmic inclusions typical of bacterial microcompartments (Fig. 3) in the majority of cells imaged. Although Eut microcompartments have been extensively imaged from Salmonella enterica serovar Enteritidis, we are not aware of previous publications showing these from uropathogenic E. coli.

Ethanolamine is not synthesized by mammals (58) and is obtained from the diet, with the ultimate source being plant and animal cell membranes. It is incorporated in phosphatidylethanolamine (PE), an aminophospholipid that is an essential constituent of cell membranes, particularly those of mitochondria and the endoplasmic reticulum (58). The source for ethanolamine detected in urine has not been established. Cell lines *in vitro* release ethanolamine into culture medium from cell membrane turnover (59). Within the gastrointestinal tract, available ethanolamine is assumed to derive from the breakdown of phospholipid from the turnover of the epithelium and dietary phospholipid (60). There is a constant supply of ethanolamine in urine in both health and infection (Fig. 1B) (49, 50), and the source in health seems unlikely to be cell turnover in the urinary tract, because this occurs at a relatively low rate compared to that in the gastrointestinal tract. The cell membranes of neutrophils and bladder epithelial cells are additional potential sources in infected urine.

There is some evidence to regard E. coli as relatively nitrogen limited in the urinary tract because it lacks urease to metabolize the most abundant nitrogen source in urine. Induction of the high-ammonium-affinity glutamine synthase and glutamate oxoglutarate aminotransferase pathway (GS/GOCAT) for nitrogen assimilation occurs in E. coli-infected urine (55, 61).

Because ethanolamine metabolism yields ammonia and acetate (Fig. 1A), in theory it should promote E. coli growth as either a sole carbon source or a sole nitrogen source. E. coli utilization of ethanolamine as a sole nitrogen source in minimal medium has been reported at concentrations of 30 mM (33). We found that 96% of clinical UPEC strains showed the utilization of 10 mM ethanolamine as a sole nitrogen source (Fig. 2A; Fig. S3A). Contradicting the assertion that concentrations of ethanolamine below 1 mM (62) do not support the growth of E. coli, we found that 0.5 mM, the level present in urine, could sustain small amounts of E. coli growth in nitrogen-limited medium (Fig. S2 and S6A). Utilization of ethanolamine by E. coli strains as a sole carbon source *in vitro* is reported to require a high ethanolamine concentration (1 g liter^−1^, 82 mM) (63). Even at this concentration, some strains showing active ethanolamine metabolism, for example, the O157:H7 EHEC strain EDL933, have been reported to be unable to use ethanolamine as a sole carbon source (33). Likewise, we found no *in vitro* growth promotion of known ethanolamine-metabolizing UPEC strains by 10 mM ethanolamine in carbon-limited minimal medium (Fig. S3B). However, in artificial urine medium (AUM), where the nitrogen sources are urea and ammonia and the carbon sources are amino acids, lactate, and citrate (64), ethanolamine at 10 mM and 0.5 mM (Fig. 2B and 4B and C; Fig. S6B) promoted additional growth of E. coli.

In M9 nitrogen-limited medium, the phenotype of *eutE* mutants showed that the ammonia liberated by the first reaction in ethanolamine metabolism, catalyzed by *eutBC* (Fig. 1A), was sufficient for growth (Fig. 4B; Fig. S6A). However, this was not sufficient for growth stimulation by ethanolamine in AUM, where *eutE* was also required (Fig. 4D), suggesting that the generation of acetyl-CoA as an additional carbon source was responsible for additional growth in this medium. A second pathway for ethanolamine conversion to acetyl-CoA has been predicted (but not defined) in Salmonella enterica from the ability of *eutBC* mutants to grow on ethanolamine as a carbon source in the presence of concentrations of carbon dioxide sufficient to change the intracellular pH (31), but no carbon dioxide was provided in our experiments.

The observation that ethanolamine at 10 mM confers a competitive growth advantage on a wild-type UPEC strain cocultured with Δ*eutE* and Δ*eutB* mutants in artificial urine medium (Fig. 5) also supports a role for acetyl-CoA generation in growth enhancement, because the extracellular acetate or ammonia deriving from wild-type cells metabolizing ethanolamine is apparently insufficient to confer growth enhancement on mutants in this medium. In contrast, E. coli strains engineered for the enhanced take-up of amino acids to grow faster on amino acids than a wild-type strain when cultured in isolation lose any growth advantage in coculture with the wild type (65). This is because the extracellular ammonia leak from enhanced amino acid metabolism in the engineered strains provides nitrogen to the wild-type strain (65).

Although we did not demonstrate a competitive advantage of wild-type E. coli over *eut* operon mutants in coculture in a physiological ethanolamine concentration of 0.5 mM (Fig. S7), this may well be due to methodological limitations. Following a 4-h lag period, ethanolamine is removed from AUM by E. coli at a rate of approximately 0.75 mM per hour (Fig. 2D), so any selective advantage due to 0.5 mM ethanolamine must be necessarily brief and difficult to detect in a competition assay based on batch culture. However, *in vivo*, host-derived ethanolamine would be continuously passing into urine at the same time as bacterial ethanolamine catabolism. The level of ethanolamine seen in noninfected urine is maintained in infected urine (Fig. 1B) containing large numbers of E. coli bacteria with induced *eut* operons (Fig. 1C and D), suggesting that it is an equilibrium level. The assertion that concentrations of ethanolamine below 1 mM (62), the level present in urine, do not support the growth of E. coli is contradicted by our *in vitro* data in both minimal medium, where ethanolamine functions as the sole nitrogen source (Fig. S6A), and the complex AUM, where it appears to function as a carbon source in addition to amino acids (Fig. 6B). Ethanolamine in urine is an important nutritional resource that infecting uropathogenic E. coli bacteria can access to augment their growth by microcompartment-mediated metabolism. These conserved metabolic pathways and structures distinct from the host offer opportunities for detection and treatment of infection.

## MATERIALS AND METHODS

### Bacterial strains and culture conditions.

Clinically infected urine samples received at Cork University Hospital (CUH) containing visible bacteria and white cells were selected and anonymized. The protocol was approved by the Clinical Research Ethics Committee of the Cork Teaching Hospitals [reference ECM 4 (c), 12 August 2014]. A further 12 specimens of macroscopically clear urine with no bacteria or white cells were selected as controls. Following initial culture on cystine-lactose-electrolyte-deficient (CLED) agar, pure colonies subcultured on Columbia blood agar were identified by matrix-assisted laser desorption ionization–time of flight (MALDI-TOF) mass spectrometry using a Microflex LT mass spectrometer (Bruker Daltonik) and the MALDI Biotyper software package (version 3.0). Antimicrobial sensitivity was determined by the Vitek (version 2.0) system (bioMérieux) using EUCAST breakpoints. The strains used for gene inactivation or competitive growth assays are listed in Table 1. Sixty-one E. coli strains were isolated, and whole-genome sequences were obtained for 47 strains.

The E. coli strains were routinely cultured in LB broth at 30°C or 37°C with aeration. To determine the ability to utilize ethanolamine, strains were cultured at 37°C in modified M9 minimal medium (33) containing 10 mM ethanolamine hydrochloride and 200 nM cobalamin with the addition of either 20 mM glycerol or 20 mM ammonium chloride. Automated growth count cultures were incubated in 96-well plates in triplicate, and the optical density at 600 nm (OD_600_) was measured using a BioTek Eon microplate spectrophotometer over 48 h. Manual growth curves were measured in 35-ml volumes with spectrophotometric analysis of 1-ml aliquots.

### Competition experiments.

Competition experiments were carried out in a published liquid artificial urine medium (AUM) (64) and with the same medium with added ethanolamine hydrochloride at 0.5 mM and 10 mM, with cell counts being performed on LB agar. Precultured E. coli strains were incubated in LB with antibiotics where appropriate. The cultures were washed in phosphate-buffered saline (PBS) and resuspended in AUM. Approximately equal concentrations of the wild type and the isogenic mutant were used to inoculate AUM with ethanolamine, as indicated in the text, to give an approximate starting OD_600_ of 0.1. The cocultures were incubated at 37°C with aeration, and at each time point the coculture was diluted 10-fold in PBS and plated onto LB agar. The dilutions were plated onto LB agar and onto LB agar containing kanamycin to determine the concentration of each strain of E. coli. The plates were incubated overnight at 37°C, and the number of CFU was calculated. The number of wild-type CFU was calculated by subtracting the number of CFU resistant to kanamycin from the number of CFU on LB agar plates. The experiment was repeated three times, and a competitive index (CI) was calculated as follows: CI = [(number of CFU of *eut* mutant recovered/number of CFU of wild type recovered)/(number of CFU in *eut* mutant inoculum/number of CFU in wild-type inoculum)]. A competitive index below 1 indicates that the wild type outcompeted the mutant strain at that time point. The CI at time zero is, by definition, 1.0. The growth of the *eut* operon mutants was compared with that of the wild-type strains in M9 minimal medium with 0.5 mM and 10 mM ethanolamine and AUM with 10 mM ethanolamine.

### Mutants.

To generate deletion mutants, E. coli BW25113 strains with knockouts of the genes of interest were obtained from the Keio Collection (66). Mutations were transferred to UPEC strain U1 by P1*vir* phage transduction (67). In brief, lysogen strains were prepared by incubating P1 lysate with the donor strain for 30 min at 30°C with 5 μl of 1 M CaCl_2_, and the culture was plated on kanamycin selective agar. The resulting colonies were used to prepare the lysate for transduction. Lysogen colonies were grown overnight in 2 ml of LB at 30°C. The precultures were used to inoculate LB and grown until they reached an OD_600_ of 0.2. The cultures were incubated at 46°C for 20 min with shaking before being moved to 37°C until complete lysis. The bacteria were centrifuged out of the culture, and the supernatant was stored with chloroform to prevent bacterial growth. Overnight cultures of the recipient strain were resuspended in transduction buffer (10 mM MgSO_4_, 5 mM CaCl_2_), and 100 μl of cells was incubated with lysate and incubated at 37°C for 30 min. Sodium citrate was added following this incubation, and the culture was incubated for a further hour. The cells were washed in LB before being plated onto LB agar plates. Strains were selected for kanamycin resistance, and transductants were confirmed by genome sequencing and PCR using primers internal to the kanamycin gene and regions upstream and downstream of the disrupted gene (see Table S1 in the supplemental material). Complementation was with E. coli K-12 genes cloned in pCA24N from the ASKA library (68) and induced by 0.01 mM IPTG (isopropyl-β-d-thiogalactopyranoside).

### Metabolic assays.

After culture, residual urine samples were separated into the cell fraction and the cell-free supernatant by differential centrifugation, and urine supernatants were filtered through a 0.2-μm-pore-size membrane to remove any remaining bacteria and stored at −80°C. Urine supernatants and culture supernatants were assayed for ethanolamine, acetate, and ethanol by HPLC using an Agilent 1200 HPLC system with a refractive index detector. Urine samples collected from CUH and bacterial culture supernatants were filter sterilized through a 0.2-μm-pore-size membrane to remove bacteria, before being stored at −80°C until the day of experimentation. Ethanolamine was measured by gradient HPLC after derivatization with *o*-phthaldialdehyde (OPA) using a method adapted from that of Sturms et al. (69). The mobile phase consisted of buffer A (10% methanol [Sigma-Aldrich], 90% 10 mM Na_3_PO_4_ [pH 7.3; Sigma-Aldrich]) and buffer B (80% methanol, 20% 10 mM Na_3_PO_4_ [pH 7.3]). Samples were prepared using an in-loop derivatization reaction, where 6 μl of sample was taken up, followed by 6 μl 10-mg/ml OPA and 3-mercaptopropionic acid in 0.4 M boric acid (Agilent Technologies), and the mixture was incubated at room temperature for 3 min. The samples were injected into a 4.6- by 100-mm, 2.7-μm-pore-size Infinity Lab Poroshell HPH-C_18_ column (Agilent Technologies) and eluted with 5 ml of a linear gradient from 50% buffer B to 100% buffer B, followed by 5 ml of 100% buffer B, at a constant flow rate of 1 ml min^−1^. The excitation was detected at 224 nm. A standard curve was created before each sequence run. Identification of the peak and quantification were determined by comparison to the retention time and the standard curve, respectively.

Acetate and ethanol were measured by the same HPLC system. Ten microliters of sample was injected into a Rezex 8-μm-particle size 8% H organic acid column (Phenomenex, USA) and eluted with 15 ml of 0.01 M H_2_SO_4_ at a flow rate of 0.6 ml min^−1^. The column was maintained at 65°C for the duration of the experiments. The identification of the substrate was determined by comparison of the retention time to that of the pure compound, and concentrations were quantified by comparison to known standards.

### TEM.

Transmission electron microscopy (TEM) was carried out as previously described (70). After growth (as indicated in the text), bacterial cells were pelleted by centrifugation to give a pellet no larger than 100 μl in volume. The bacterial pellet was fixed in 2 ml of 2.5% glutaraldehyde (Fluka) diluted in 0.1 M sodium cacodylate (CAB), pH 6.8 (bioWorld). After incubation overnight at 4°C, the bacteria were washed twice with 0.1 M CAB and suspended in 2 ml of fresh 2.5% glutaraldehyde diluted in CAB. The bacteria were stained for 1 h in 1 ml of 1% (wt/vol) osmium tetroxide (250 μl 4% osmium tetroxide, 250 μl Milli-Q H_2_O, 500 μl 0.2 M CAB). The pellets were washed in 2 ml Milli-Q H_2_O for 10 min twice before the pellets were dehydrated. The pellets were dehydrated through an ethanol (EtOH) gradient as follows: 50% (vol/vol) EtOH for 10 min, 70% EtOH for 10 min, 90% EtOH for 10 min, and 100% EtOH for 10 min three times. The bacterial pellets were then washed twice in propylene oxide for 10 min. The pellets were embedded in 1.5 ml propylene oxide–low-viscosity (LV) resin at 1:1 for 30 min, followed by incubation 2 times for 1.5 h each time in 100% freshly made agar LV resin. The pellets were resuspended in 1 ml of 100% LV resin and transferred to a conical-bottom tube. The bacterial pellet was centrifuged at 1,100 × *g* for 5 min and was left to incubate at 60°C for 24 h. Bacteria were sectioned to 60 to 70 nm with a diamond knife on a Leica EM-UC7 ultramicrotome. Sections were collected on 400-mesh copper grids and stained with 4.5% (wt/vol) uranyl acetate in 1% (vol/vol) acetic acid for 45 min and Reynolds lead citrate for 7 min at room temperature. Sections were then observed on a JEOL 1230 transmission electron microscope operated at an accelerating voltage of 80 kV and imaged with a Gatan OneView digital camera.

### DNA sequencing, sequence analysis, and statistics.

DNA was extracted from overnight cultures in LB and extracted using a Qiagen DNeasy blood and tissue kit (Qiagen) with RNase A treatment (Sigma). Bacterial genome sequencing was carried out by MicrobesNG (see Acknowledgments) using Illumina HiSeq 2500 2 × 250bp paired-end reads. Reference genomes were identified using a Kraken system (71), and reads were mapped using the BWA-MEM algorithm (72). *De novo* read assembly was achieved using the SPAdes algorithm (73), with read mapping back to the resultant contigs, using the BWA-MEM algorithm for quality metrics. Automated annotation was performed using Prokka software (74).

Phylogenetic trees were generated from contig sequences with Parsnp (Harvest tool suite [36]) and edited with the iTOL tool (75). Parsnp produces a core genome alignment and identifies SNPs for tree generation by the FastTree (version 2) tool (76) using Shimodaira-Hasegawa (SH)-like local supports for bootstrapping. Alignment with 32 reference genomes known to be representative of six E. coli phylogroups (77) was used for phylogroup assignment. Gene presence in genomes was taken as >75% identity in a BLASTN search over the full reference gene sequence length. Binary matrices representing sequenced genomes were prepared, with PUF gene presence scored as 1 and absence scored as 0 and phenotypic antimicrobial resistance scored as 1 and sensitivity scored as 0. Two-dimensional cluster analysis on these matrices was performed with the R software package using complete linkage clustering on the Jaccard distance. The resulting cladograms and heat maps were visualized with the iTOL tool (75). All other statistical analyses presented were generated with GraphPad Prism (version 7) software.

### RNA and RT-PCR.

RNA was extracted from bacterial pellets using a Zymo fungal/bacterial miniprep kit and from eukaryotic cells using a Quick-RNA miniprep kit, following the manufacturer’s instructions. After extraction, genomic DNA was digested using Turbo DNA-free (Ambion) DNase I treatment. The RNA was quantified using a NanoDrop 1000 spectrophotometer. cDNA was synthesized by reverse transcription, carried out in nuclease-free 96-well plates. RNA was diluted using molecular-grade H_2_O (Sigma-Aldrich) to a final concentration of 100 ng μl^−1^ in a 10-μl volume. The RNA was mixed with the cDNA reaction mixture, which was set up with 4 μl 5× reverse transcription buffer (Roche), 3 μl random hexamer primer (Roche), 2 μl 20 mM deoxynucleoside triphosphate mix, and 1 μl reverse transcriptase/RNase inhibitor to give a total volume of 20 μl. The reaction mixture was incubated in a thermocycler under the following conditions: 10 min at 25°C, 30 min at 55°C, and 5 min at 85°C, with a hold at 4°C. The cDNA was then diluted to 100 μl and stored at −20°C until use.

The universal probe library (Roche, Indianapolis, IN, USA) was utilized to design primers for quantitative PCR. The primers used in this study are listed in Table S1. Amplification reaction mixtures were a mix of 3 μl of cDNA, 7 μl TaqMan probe master buffer (Roche), 1 μl 20 mM primer pair mix, 0.1 μl probe (Roche), and 0.9 μl molecular-grade H_2_O to a make a final volume of 10 μl. When the probe was not available, a SYBR green master mix was used and included 3 μl cDNA, 5 μl 2× SYBR green I master buffer (Roche), 1 μl 20 mM primer mix (L+R primers), and 1 μl molecular-grade H_2_O to a final volume of 10 μl. All reactions were performed using a 384-well plate on a LightCycler 480 system (Roche), with molecular-grade water included as a negative control. Thermal cycling conditions were as follows: 50°C for 2 min and 95°C for 10 min, followed by 45 cycles of 95°C for 10 s, 60°C for 45 s, and 72°C for 60 s. Relative gene expression was calculated using the 2^−ΔΔ^*^CT^* threshold cycle (*C_T_*) method (78). The fold change in the amount of mRNA of the target gene was quantified relative to the amount of the *gyrA* gene.

### ELISA.

Frozen urine samples were analyzed using Meso Scale Discovery (MSD) V-Plex proinflammatory panel I and cytokine panel II (MSD, Rockville, MD) enzyme-linked immunosorbent assays (ELISAs). Assays were performed according to the manufacturer’s instructions, and concentrations were measured using a Meso QuickPlex SQ120 instrument. Calibrators were run in duplicate with the urine samples and used to form a standard curve. The concentrations of cytokines in the urine were extrapolated from the standard curve. Values which fell below the limits of detection were excluded from statistical analysis.

### Accession number(s).

Sequencing data are available for download from the EBI European Nucleotide Archive under BioProject accession numbers PRJEB31941, PRJEB31942, PRJEB31943, and PRJEB31944.

## Supplementary Material

Supplemental file 1
