# Supplementary material for: Bacterial Microcompartment-Mediated Ethanolamine Metabolism in Escherichia coli Urinary Tract Infection
Source: Infect Immun. 2019 Jul 23;87(8):e00211-19. doi: 10.1128/IAI.00211-19 (PMC6652756; doi:10.1128/IAI.00211-19)
Supplement: Supplemental file 1 [file IAI.00211-19-s0001.pdf]

Supplementary Data: Dadswell et al

Table of Contents

Table of Contents	Page
**Tables**	
**Table S1. Primers used in this study**	**2**
**Table S2. Competitive index of *eut* operon mutant strains grown in co-culture with the wild-type in AUM with 10mM ethanolamine.**	**3**
**Supplementary Figures**	
**Figure S1. Proinflammatory cytokines detected in infected urine.**	**4**
**Figure S2. Utilisation of ethanolamine as a nitrogen source in minimal medium by urine isolates of *E.coli***	**5-7**
**Figure S3. Comparison of utilisation of ethanolamine as a nitrogen and carbon source for selected urinary isolates of E. coli in a microtitre plate assay**	**8**
**Figure S4. RT-PCR shows upregulation of the *E. coli* U1 *eut* operon in AUM with addition of ethanolamine and does not show polarity of single gene *eutB* or *eutE* mutants**	**9**
**Figure S5. Scanty bacterial microcompartments are visible in *E. coli* U1 when grown in M9 with ethanolamine**	**10**
**Figure S6. *In vitro* growth advantage of UPEC strain U1 conferred by physiological levels of ethanolamine**	**11**
**Figure S7. Inactivation of *eut* operon genes does not reduce competitiveness of *E. coli* UPEC strain U1 in artificial urine medium containing 0.5 mM or less ethanolamine**	**12**
**Figure S8. Acetate is present in infected urine**	**13**

Table S1. Primers used in this study

Name	Sequence 5'-3'	Source
**Colony PCR Primers**		
KD13_K1	CAGTCATAGCCGAATAGCCT	Baba et al (1)
KD13_K2	CGGTGCCCTGAATGAACTGC	Baba et al (1)
eutR_L	TTGTTGGCGCTGTTAACATC	This study
eutR_R	GGCGGGCTGTTCTTTGATGA	This study
eutB_L3	TACCCTTTCGCTCTCTGGCAG	This study
eutB_R3	AGTGATCGGCCAGGAAACGCA	This study
eutE_L	ATTGTCGATGAGGTGGTGTC	This study
eutE_R	TGGTTACACAGCGTTGCC	This study
eutRin_L	TCGTTCTGGTTTGGCATTCC	This study
eutRin_R	GACGGCTTCTCGGAAAACAG	This study
eutBin_L	ATGTGCTGAGCGATGAAACC	This study
eutBin_R	CCCATGATGTAGTTGCAGCC	This study
eutEin_L	ATTGCTGCCATTCGTGAAGC	This study
eutEin_R	ATGTTCTGCGGTGGTTTCCA	This study
**qPCR Primers**		
EC_gyrA_L	GCAGGATCGCAGTGATAGC	This study
EC_gyrA_R	GTCGTCCGATCGTCAACC	This study
EC_rrsA_pL5_L	GACGATCCCTAGCTGGTCTG	This study
EC_rrsA_pL5_R	CGTAGGAGTCTGGACCGTGT	This study
EC_eutB_pL_34_L	CGCGGAGTTCAACCGTAT	This study
EC_eutB_pL_34_R	CAGAGCCTTGTCCGGTTTC	This study
EC_eutR_pL_74_L	TGTCTATGAACATGCTTTAACGATT	This study
EC_eutR_pL_74_R	CGGATGCAGCTGATCGTA	This study
EC_eutS_pL_153_L	CACACTGGCGCATCTCAT	This study
EC_eutS_pL_153_R	CCGGGAGTTAGCGTCATAAT	This study
EC_eutE_pL_23_L	AGAACGGACCGTGCATTG	This study
EC_eutE_pL_23_L	GGTGATGGTCATGGTGGTC	This study

Table S2. Competitive index of *eut* operon mutant strains grown in co-culture with the wild-type in AUM with 10mM ethanolamine.

Co-cultured Strains	Incubation time (Hours)						
	**12**	**24**	**24(I)**	**28**	**32**	**36**	**48**
U1/U1ΔeutB	0.51	0.72	0.42	0.52	0.19	0.42	0.40
U1/U1ΔeutE	0.36	0.48	0.36	0.40	0.28	0.24	0.26

CI is calculated as described in Methods and Supplementary Methods. All values are below 0.8. 24 (I) indicates that the bacteria were subcultured. Values are Means of N=3.

Supplementary Figures

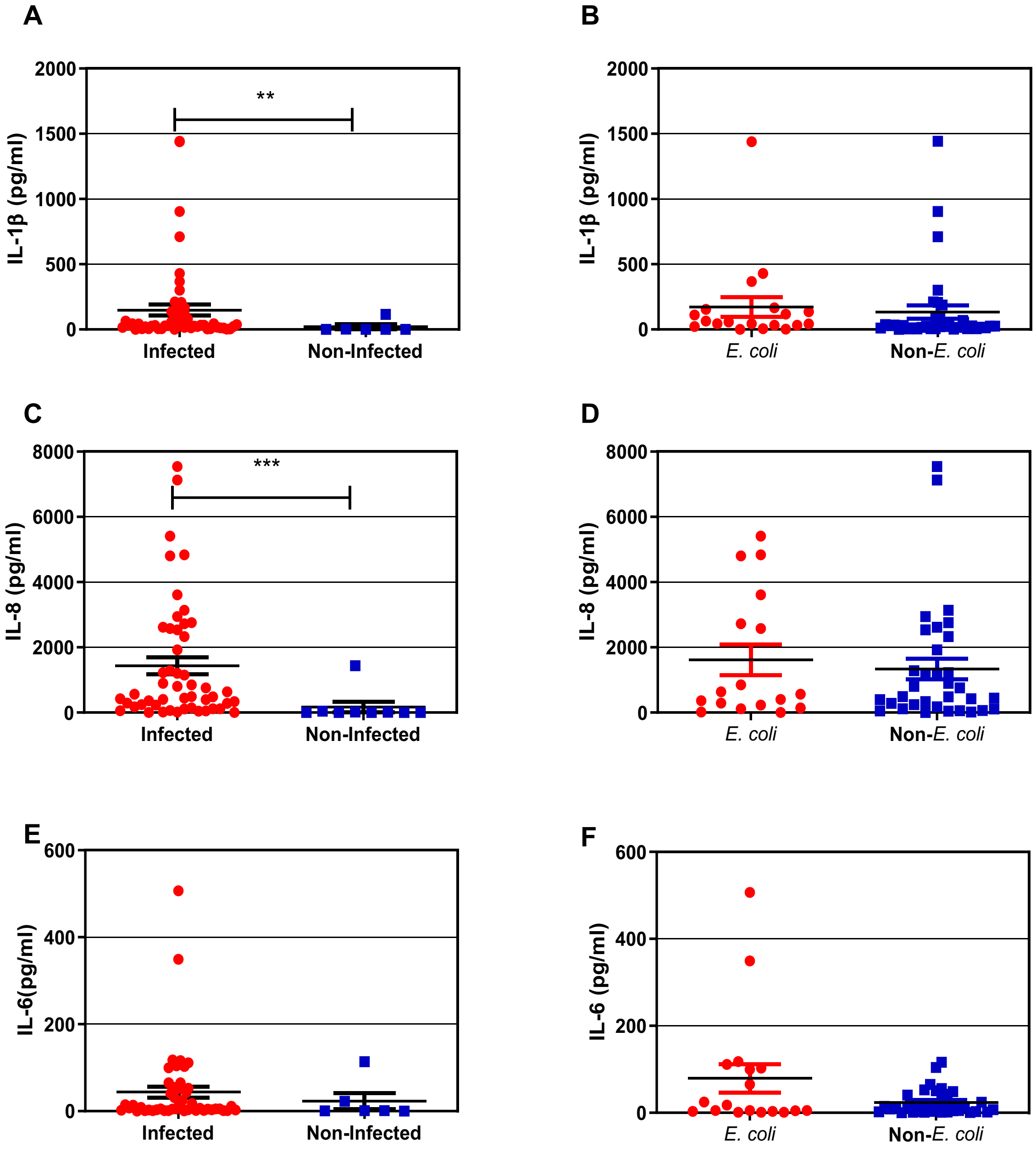

Figure S1. Pro-inflammatory cytokines detected in infected urine
A,B IL-1β **C,D** IL-8 **E,F** IL-6. **A,C,E** infected urine (n=52) compared to non-infected urine (n=12). **B,D,F** *E. coli* infected urine (n=19) and non *E .coli* infected urine (n=28). Compared by Mann Whitney-U ** $p \leq 0.01$ *** $p<0.001$

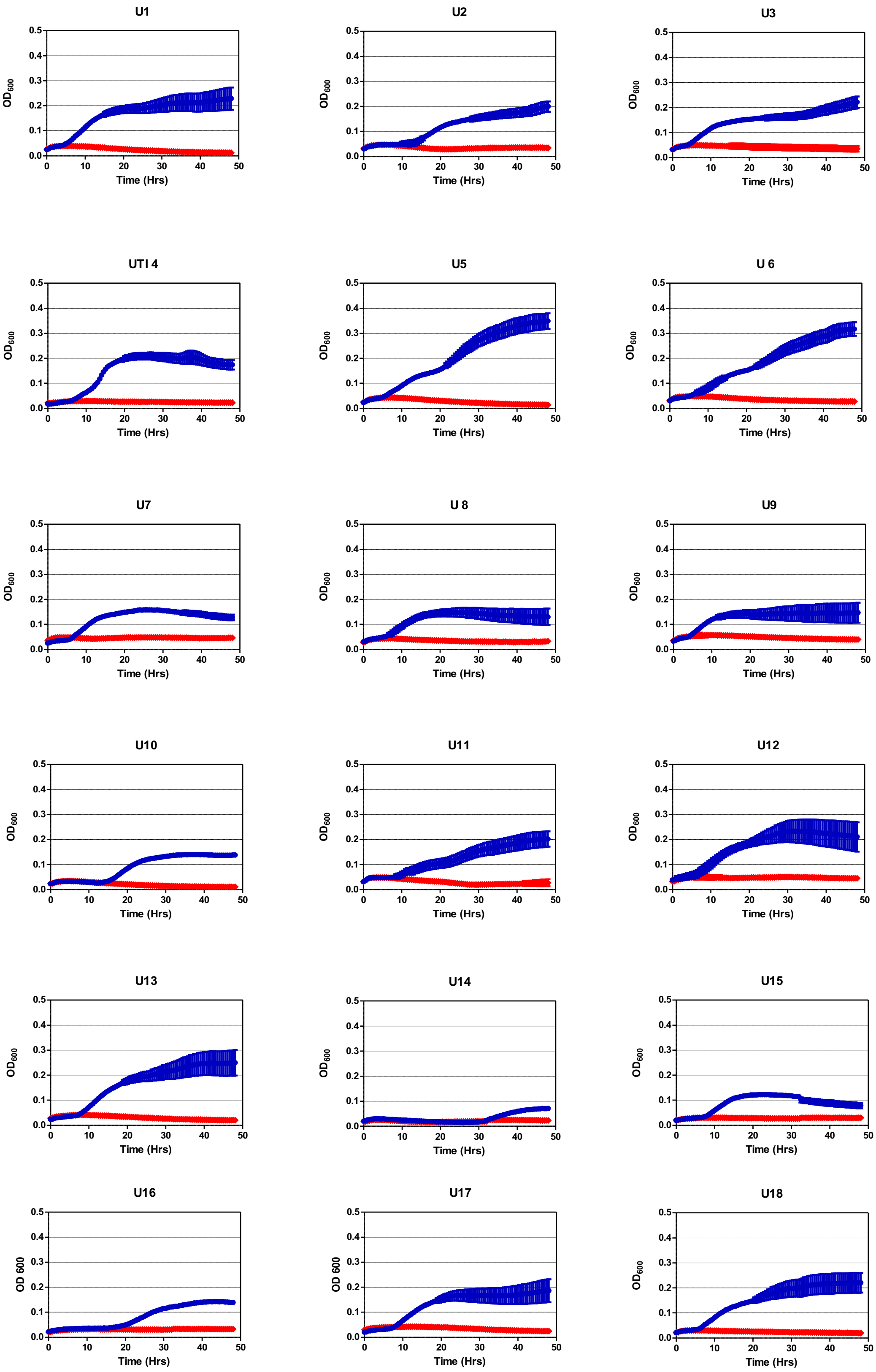

Figure S2. Utilisation of ethanolamine as a nitrogen source in minimal medium by urine isolates of *E. coli*

Automated growth curve of UPEC isolates U1-U79 grown in ammonia-free modified M9 (red) and ammonia-free modified M9 supplemented with 10mM Ethanolamine as nitrogen source. Values are Mean ± SEM. N=3.

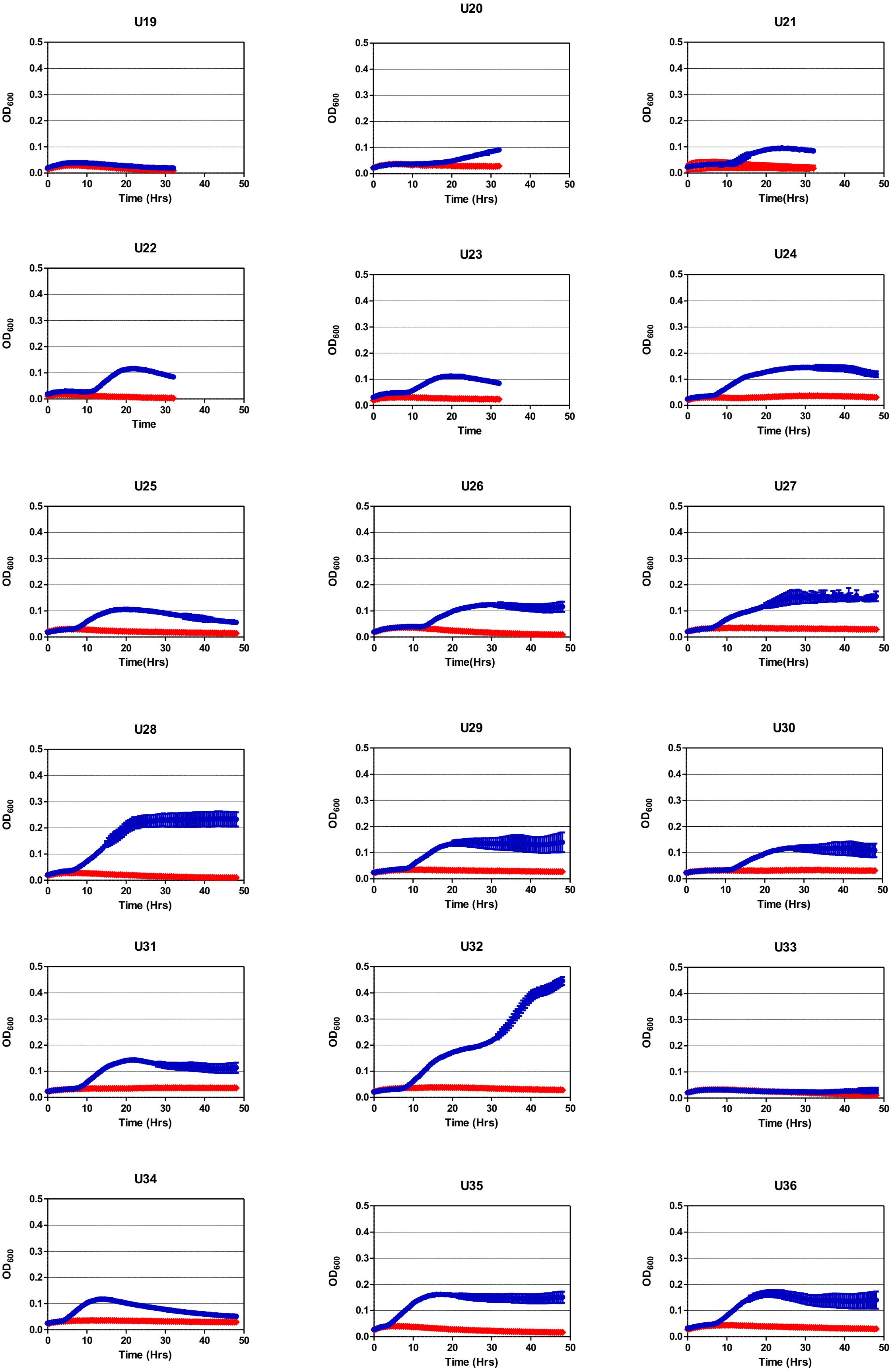

Figure S2 (continued). Utilisation of ethanolamine as a nitrogen source in minimal medium by urine isolates of *E. coli*

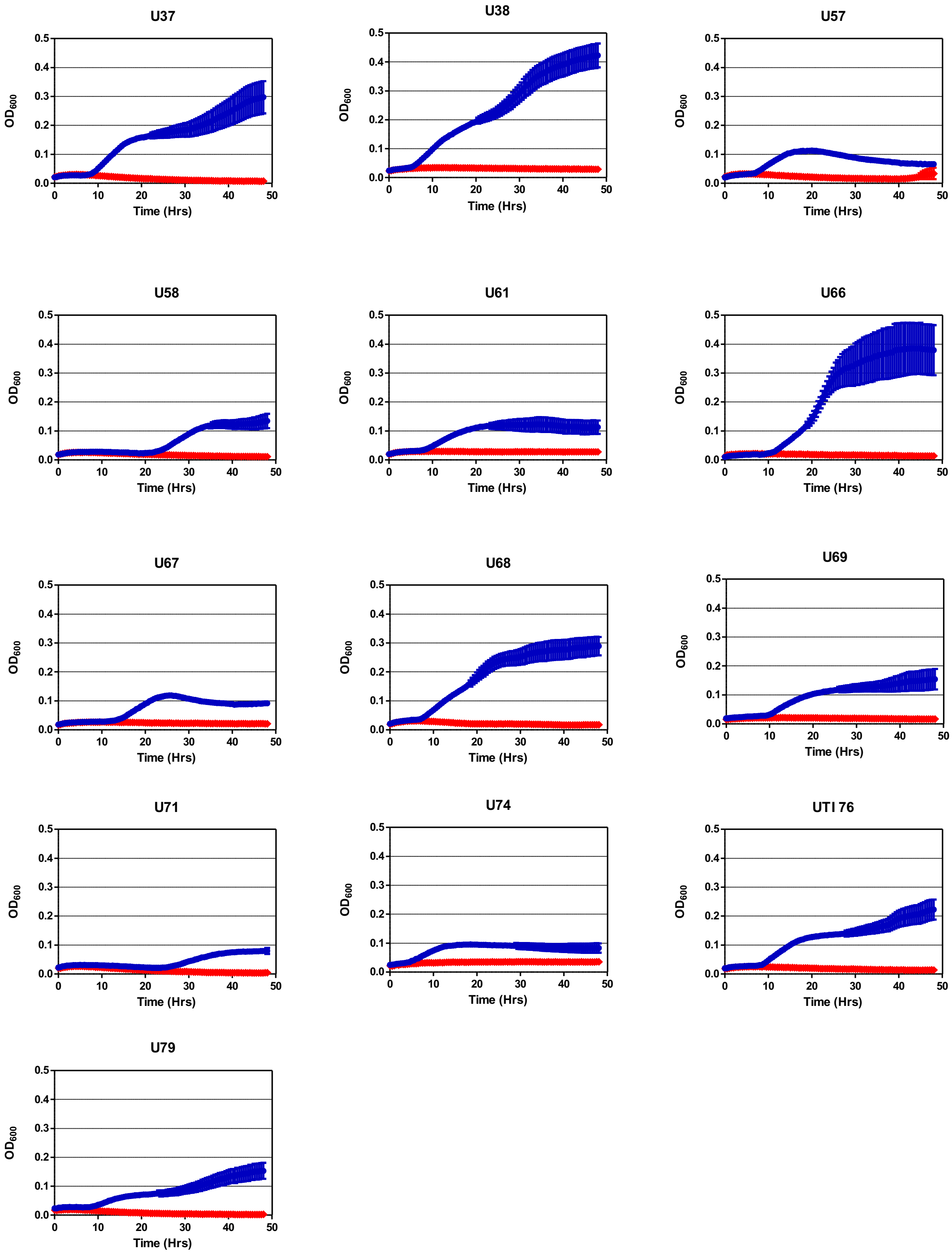

Figure S2 (continued). Utilisation of ethanolamine as a nitrogen source in minimal medium by urine isolates of *E. coli*

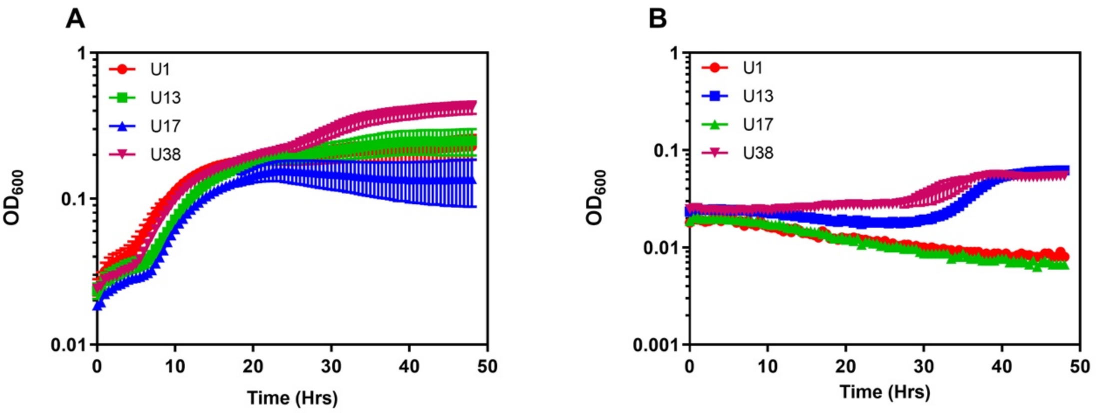

Figure S3. Comparison of utilisation of ethanolamine as a nitrogen and carbon source for selected urinary isolates of *E. coli* in a microtitre plate assay

A. Modified M9 minimal medium with ethanolamine 10 mM, glycerol 10 mM. B. Modified M9 minimal medium with ethanolamine 10 mM, ammonium chloride 10 mM.

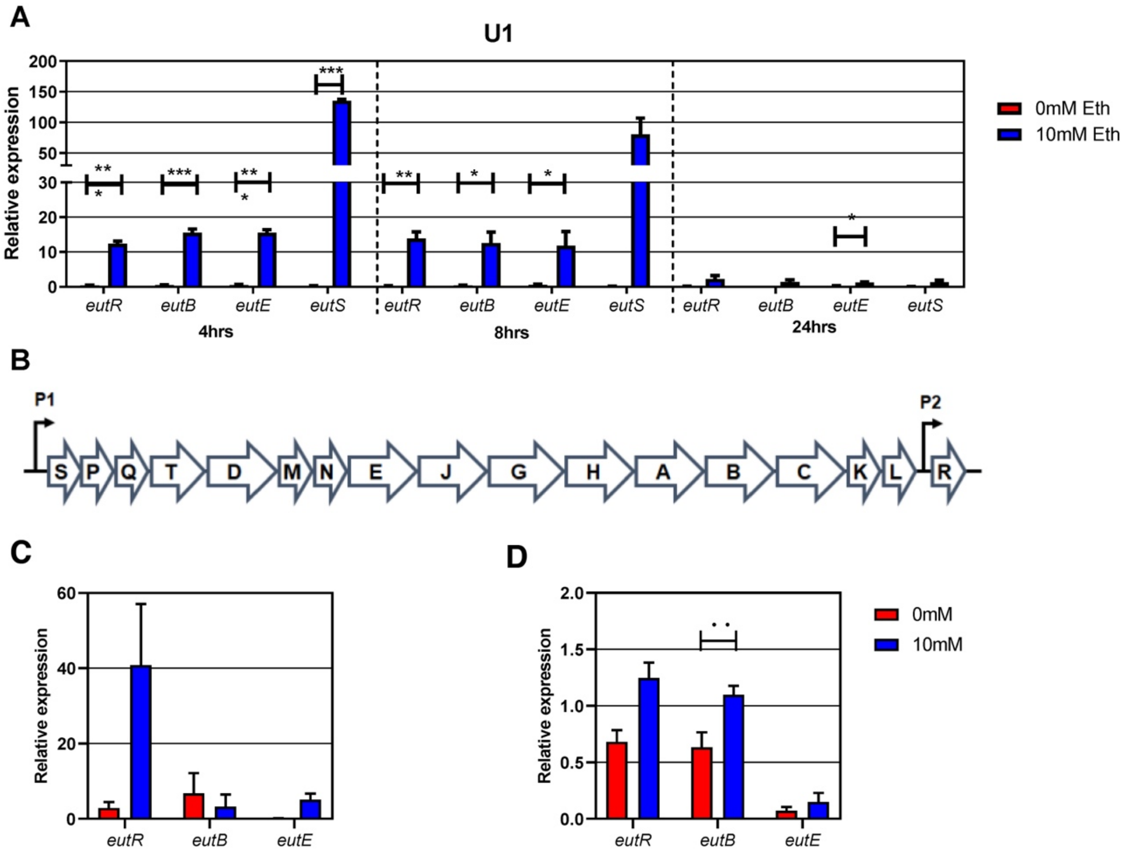

Figure S4. RT-PCR shows upregulation of the *E. coli* U1 *eut* operon in AUM with addition of ethanolamine and does not show polarity of single gene *eutB* or *eutE* mutants

The eut operon is upregulated on growth in AUM following the addition of 10mM ethanolamine. A. Relative expression of *eutR*, *eutB*, *eutE* and *eutS* in U1 grown in AUM with (blue) and without (red) 10mM ethanolamine. B. Schematic of the *eut* operon.
Relative expression of *eutR*, *eutB* and *eutE* in C. U1Δ*eutB* and D. U1Δ*eutE* after 4 hours growth in AUM with and without 10mM ethanolamine. Expression is measured relative to the geometric mean of *rrsA* and *gyrA*. Values are Mean ± SEM. N=3. * p<0.05, **p<0.01, Mann-Whitney U-test.

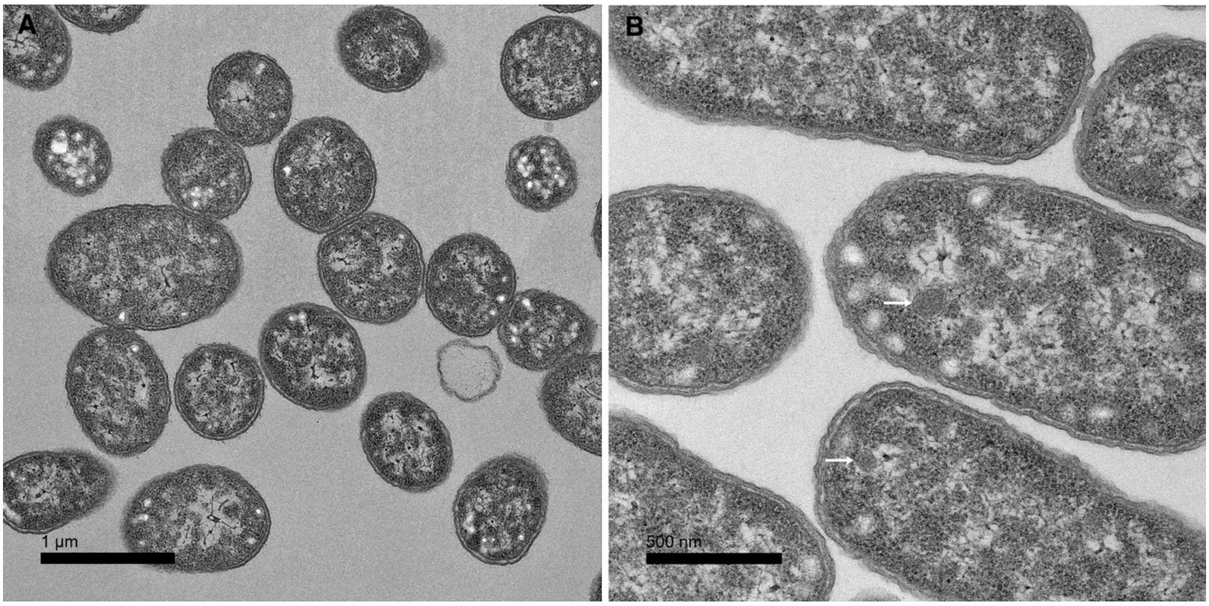

Figure S5. Scanty bacterial microcompartments are visible in *E. coli* U1 when grown in M9 with ethanolamine
Transmission electron microscopy of cells grown in modified M9 medium with 20 mM - glycerol for 8 hours. A. U1 in modified M9 with 20 mM ammonium chloride. B. U1 grown in modified M9 with 10mM ethanolamine. Arrows indicate microcompartments.

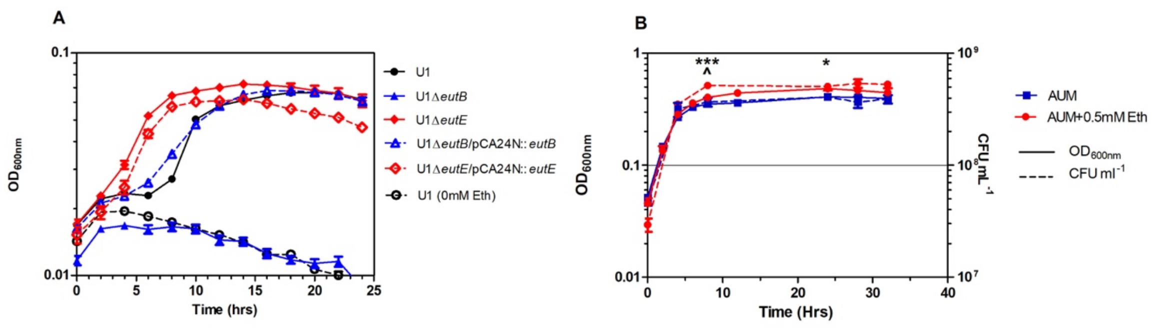

Figure S6. ***In vitro*** **growth advantage of UPEC strain U1 conferred by physiological levels of ethanolamine**

A. Nitrogen-limited M9 minimal medium with 0.5 mM ethanolamine 20 mM glycerol, control U1 growth without ethanolamine black dotted line. B. AUM (blue) and AUM with 0.5 mM ethanolamine (red). Solid lines OD_{600} measurements, dotted lines CFU ml^{-1} counts. Significant difference in OD_{600nm} *** $P<0.001$ * $P<0.05$. Significant difference in CFU ml^{-1} ^ $P<0.05$.(Student's t-test)

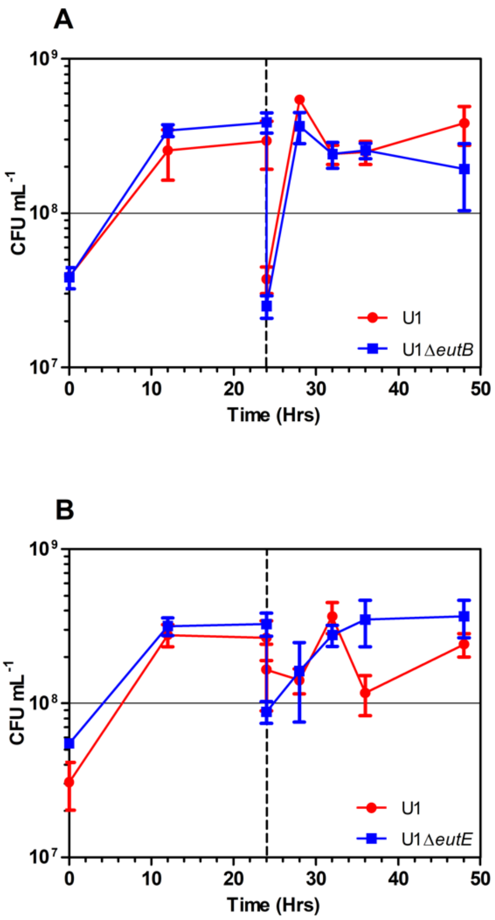

Figure S7. Inactivation of *eut* operon genes does not reduce competitiveness of *E. coli* UPEC strain U1 in artificial urine medium containing 0.5 mM or less ethanolamine
Competition of U1 vs A. U1Δ*eutB* with 0.5mM ethanolamine B. U1Δ*eutE* with 0.5mM ethanolamine.Vertical dotted line marks subcultures to fresh media at 24 hours. Values are Mean ± SEM. N=3.

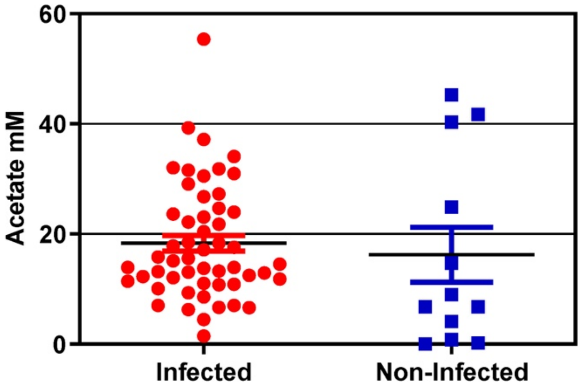

Figure S8. Acetate is present in infected urine
Red infected urine, blue non-infected. HPLC assay.
